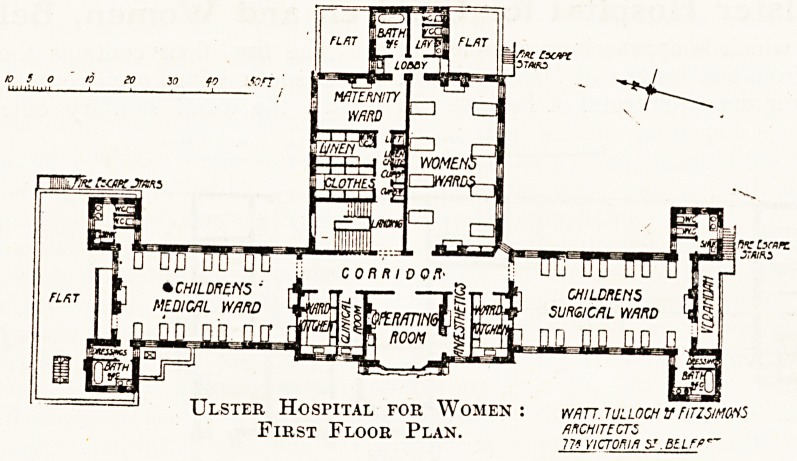# Ulster Hospital for Children and Women, Belfast

**Published:** 1912-05-11

**Authors:** 


### HOSPITAL ARCHITECTURE AND CONSTRUCTION.

[Communications on this subject should be marked "Architecture" in the left-hand top corner of the envelope.]

### Ulster Hospital for Children and Women, Belfast.

This building, which is approaching completion, is being erected from the designs of Messrs. Watt, Tulloch and Fitzsimons, architects, of Belfast. In form the building is planned like a T reversed. On the ground floor the whole of the north wing is taken up with the out-patient department. This comprises a large waiting-hall, a consulting-room and dressing-room for women, a medical and surgical consulting-room, presumably for children, with operating-room and recovery-room adjoining, a dressing-room, and an ophthalmic consulting-room with dark-room attached. The dispensary is placed at the extreme south-east angle of the south wing, and to reach it out-patients would have to pass along the whole length of the ground-floor corridor giving access to the staff sitting-rooms and the board-room. This seems a remarkably inconvenient arrangement.

The main entrance, with waiting-room, occupy the centre of the front, and on one side is the matron's sitting-room, and on the other, sisters' and nurses' sitting-rooms and board-room, with district-nurses' store entered from the outside.

At the back is the staff dining-room and the kitchen offices. Coal and wood stores and an open shed are placed round an open yard at the back, and a small mortuary adjoins the latter. A small detached building contains an isolation ward and nurses' room. This is really an observation ward, and is only intended for the reception of cases of doubtful diagnosis, pending their transfer either to the wards or the fever hospital.

Ulster Hospital for Women: Ground Floor Plan.

WATT. TULLOCH & FITZSIMONS ARCHITECTS 77A VICTORIA ST. BELFAST.

The first floor contains the wards. The north and south wings each contain a ward for 15 beds, with the usual sanitary offices in angle towers. In the centre is the operating-room, having on one side a small anæsthetic-room, and on the other side a small clinical-room. To each ward is attached a ward kitchen. The back building is bisected by a wall; the southern half is occupied by a ward for eight women, while the northern half contains

SECOND FLOOR PLAN

the staircase, rooms for the storage of linen and patients' clothes, a lift, a chute for soiled linen, cupboards, and a maternity ward. The sanitary offices for the large ward and the maternity ward are built out at the back and are used in common.

This part of the building is open to criticism on several points. The large ward has windows on one side and a window at one end, so that cross-ventilation is practically non-existent. The access to the maternity ward is by a narrow and seemingly dark passage into which two shafts open—one being the lift, the other the chute for soiled linen. This last is a most objectionable arrangement, and may, if the greatest care is not exercised, become a serious source of mischief.

The upper floor, which includes the centre part only, is devoted entirely to bedrooms for the staff, with bath-rooms, w.c.s, stores, etc.

Ulster Hospital for Women: First Floor Plan.

## Figures and Tables

**Figure f1:**
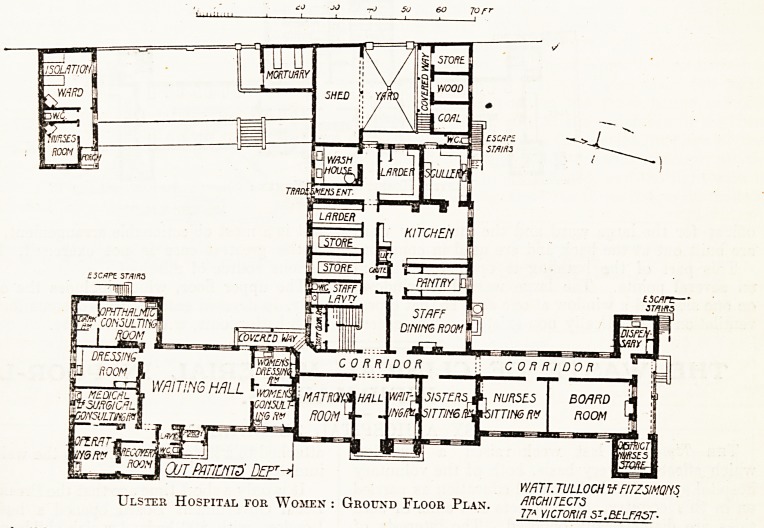


**Figure f2:**
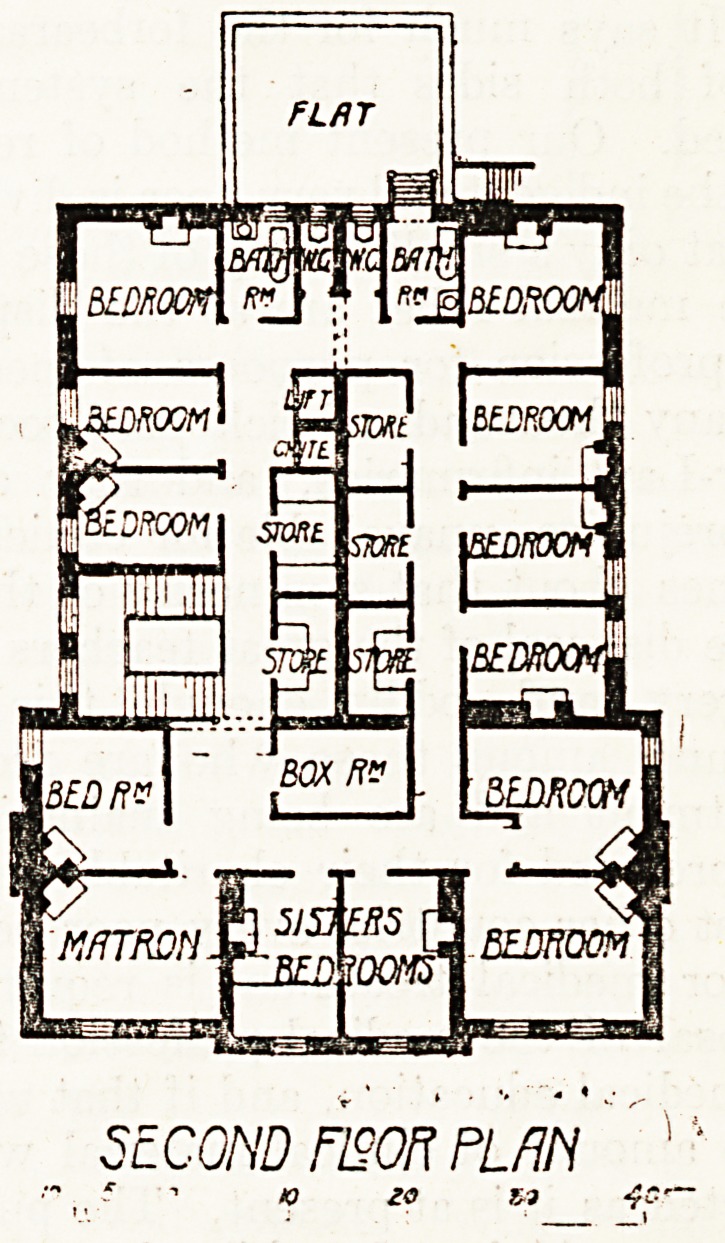


**Figure f3:**